# Effects of Oxytetracycline/Lead Pollution Alone and in the Combined Form on Antibiotic Resistance Genes, Mobile Genetic Elements, and Microbial Communities in the Soil

**DOI:** 10.3390/ijerph192315619

**Published:** 2022-11-24

**Authors:** Tengfei Guo, Zhaoyi Li, Yanqiu Shao, Yanli Fu, Weiyi Zhang, Yingying Shao, Ying Zhu

**Affiliations:** 1 Advanced Materials Institute, Qilu University of Technology (Shandong Academy of Sciences), Jinan 250014, China; 2 Shandong Nuclear and Radiation Safety Monitoring Center, Jinan 250117, China

**Keywords:** oxytetracycline, lead, antibiotic resistant genes, microbial communities, soil, mobile genetic elements

## Abstract

The application of livestock manure is the leading cause of antibiotic and heavy metal pollution in agricultural soil. However, the effects of oxytetracycline (OTC) and lead (Pb) pollution in the single or combined form on antibiotic resistance genes (ARGs) in the soil need to be further studied. This study was planned to investigate the effects of OTC and Pb application on ARGs, mobile genetic elements (MGEs), and bacterial abundance in the soil. The relative abundance of ARGs and MGEs increased by 0.31-fold and 0.03-fold after the addition of 80 mg kg^−1^ Pb to the soil, and by 0.49-fold and 0.03-fold after the addition of 160 mg kg^−1^ Pb. In addition, under the premise of the existence of OTC, the inhibitory effect of a low concentration of Pb on ARG is stronger than that of a high concentration of Pb, resulting in a lower abundance of ARGs. The abundance of ARGs and MGEs increased by 0.11-fold and 0.17-fold after the addition of OTC (30 mg kg^−1^) to the soil at a Pb concentration of 80 mg kg^−1^ and by 0.18-fold and 0.04-fold at a Pb concentration of 160 mg kg^−1^. The addition of OTC and Pb in the soil also decreased the many bacterial communities such as Bacteroidetes, Proteobacteria, Acidobacteria, and Firmicutes. Redundancy analysis (RDA) showed that organic matter content and pH were positively correlated with the abundance of ARGs and MGEs. At the same time, electrical conductivity (EC) had a negative correlation with the abundance of ARGs and MGEs in the soil. *Intl1* was significantly associated with *tetB*, *sul1*, *tetQ*, *sul2*, and *sul3*. Network analysis illustrated that Actinobacteria, Bacteroidetes, and Proteobacteria were the main host bacteria causing changes in the abundance of ARGs and MGEs, and they were also predominant phylum in the culture environment. This conclusion can provide a reference for the related research of ARGs in soil.

## 1. Introduction

In recent years, contamination of soil with antibiotic-resistant genes (ARGs) has steadily become a recently focused research topic. Many studies have presented that ARGs can be commonly detected in places such as air, soil, feces, sewage treatment plants, rivers, irrigation ditches, hospitals, etc. Nowadays, ARGs pollution has become a global issue and has significantly impacted the public health and the economy of countries [1,2,3,4,5,6,7]. The use of veterinary antibiotics and heavy metals in livestock and poultry production causes the ARGs pollution of soil through various direct and indirect means. As veterinary antibiotics and heavy metals are not fully absorbed and utilized in the body of animals, residues of these drugs excrete in the urine and feces of livestock and poultry. The livestock and poultry manure is simply treated and used as organic fertilizer for agriculture farming. However, soil contamination with heavy metals and antibiotics also occurs along with farming, indirectly changing the distribution of ARGs and affecting the structure and function of microbial communities in the soil. Furthermore, changes in the concentrations of soil pollutants can indirectly affect the selection pressure of soil bacterial communities, which further makes bacterial communities resistant to various pollutants to varying degrees [8,9,10,11,12,13,14,15,16,17,18,19,20,21,22].

A lot of research has been conducted on the impact of heavy metals and antibiotics pollution alone as well as in combination with ARGs abundance in the soil. Qi et al. found a significant correlation between heavy metals and ARGs when studying the effect of heavy metals such as Cd, As, Cu, Ni, Pb, and Zn pollution on ARGs in the soil [23]. Wang et al. investigated the abundance of ARGs in heavy metal-contaminated soils and found that heavy metals could aggravate the abundance of ARGs in the soil by increasing the abundance of metal resistance genes (MRGs) or affecting bacterial communities. Furthermore, high levels of heavy metals enhance the co-selection of ARGs and MRGs [24]. Hu et al. investigated the changes in the abundance of ARGs caused by Cu pollution, the results indicated that the abundance of ARGs was positively correlated with mobile genetic elements (MGEs), and there was a symbiotic relationship between ARGs and soil microflora [25]. Guo et al. found that Cu and Zn were positively correlated with some *tet* and *sul* genes in the investigation of heavy metals, antibiotics, and ARGs in soil [26]. Antibiotic residues in the soil also could promote the propagation of ARGs encoding defense mechanisms [27,28]. Luo et al. found that the abundance of ARGs increased in sediments after three-month of use of oxytetracycline (OTC); the integrated mechanism of horizontal gene transfer was the main cause of the spread of *tet* ARGs changes in the microbial population and ARGs in a sedimentary mariculture environment [29]. Meanwhile, Zhao et al. found that bacterial resistance decreased with the increase in antibiotic concentration. Additionally, the application of poultry manure containing antibiotic residues increased ARGs in the agricultural soil compared to the control group [30]. Guo et al. found that OTC residue in soil and soil pH were the main factors affecting the changes of ARGs and *intl1* genes, and when OTC and Cd were added to soil at the same time, the abundance of ARGs and *intl1* genes in soil would be further increased [31]. Therefore, it is concluded that the effect of heavy metals and antibiotic residues on the abundance of ARGs is mainly regulated due to changes in the bacterial community and MGEs.

Previous studies mainly focused on the effects of heavy metals such as Cu, Zn, and different antibiotic combinations on the structure of the microbial communities and ARGs [32,33,34]. However, there have been few studies on the effects of Pb and OTC contamination alone or in combination on ARGs and microbial communities in the soil. Therefore, the alone or combined effect of OTC and Pb on ARGs, MGEs, and microbial communities in the soil was conducted to investigate the effects of Pb and OTC alone and in combination on the abundance of ARGs in the soil and soil microbial communities, and to analyze the correlation between ARGs and potential host bacteria. This study contributed toward understanding the mechanism of action of Pb and OTC on ARGs and provided a theoretical basis for studying ARGs transfer in the environment.

## 2. Materials and Methods

### 2.1. Materials

The testing soil sample was taken from a poultry farm in Jinan City, Shandong Province, China (36°22′53.170″ N, 117°1′56.695″ E). The topsoil (0~20 cm) and fresh chicken manure were collected, naturally dried, crushed, filtered through a 10-mesh sieve, and then thoroughly mixed, respectively. The physical and chemical properties of the topsoil and chicken manure are listed in Table 1. Lead nitrate [Pb (NO_3_)_2_] and OTC were added to the soil as pollutants. OTC (purity > 95%) was supplied by Solarbio (Beijing, China), and [Pb (NO_3_)_2_] and Sinopharm Chemical Reagent Co. supplied other chemical reagents.

### 2.2. Experimental Design

The original topsoil sample and chicken manure were air-dried and mixed to make the test soil sample (14.3% wt/wt). The amount of test soil used in each experimental group was 180 g. The experiment was split into nine groups with identity CK, C30, C100, P80, P160, C30P80, C30P160, C100P80, and C100P160, as listed in Table 2. According to the experimental design, [Pb (NO_3_)_2_] and OTC were dissolved in water, respectively, and then evenly sprayed in the soil and fully mixed with the soil. The CK group was replaced by ultrapure water of the same volume to ensure the same water content. Each treatment was repeated three times, and all treatments were carried out for 90 days in a dark environment at 25 °C. Ultrapure water was added to the simulated soil every other day, and the maximum soil moisture of the field was maintained at 60% during the incubation period by weighing.

### 2.3. Sample Collection

A destructive sampling method was used in this study and soil samples were taken from the incubator on days 1, 7, 20, 50, and 90. After thoroughly mixing, the sample was divided into three parts, some of which were stored at 4 °C, and soil pH, organic matter, and electrical conductivity (EC) were determined within one week, as previously reported. The other samples were stored at −20 °C for subsequent experimental analysis, and the remaining samples were stored at −80 °C for the 16 S rRNA gene high-throughput sequencing. Soil pH was estimated when the soil/water ratio was 1:2.5 (*w*/*w*). The organic matter content of the soil was determined by K_2_CrO_4_ oxidation and FeSO_4_ titration method, as previously demonstrated. Soil EC was measured by a conductivity meter at a 1:5 soil: water suspension ratio (DDS-307A, Rex, Shanghai, China) [35,36,37].

### 2.4. Real-Time Quantitative PCR (qPCR)

Genomic DNA was extracted using the TIANamp Soil DNA Kit (TianGen, Beijing, China). The extracted DNA was stored at −20 °C for subsequent experimental analysis. Five ARGs (*tetB*, *sul1*, *tetQ*, *sul2*, and *sul3*), two MGEs (*intl1*, *intl2*), and 16 S rRNA were identified by using gene primers listed in Table 3.

### 2.5. 16 S rRNA Gene High-Throughput Sequencing

Illumina NovaSeq platform was used to sequence the qPCR product. The abundance of sample microbial species was analyzed by Alpha abundance analysis by using QIIME2 software (version 2019.7, San Diego, CA, USA).

### 2.6. Data Processing

Spearman’s correlation coefficient was calculated by the SPSS computer program version 25.0 (IBM, Armonk, NY, USA). Network analysis, redundancy analysis, and heat map analysis were drawn with the OmicStudio tool, and the charts were made using Microsoft Excel (2019) and Origin (version 2020).

## 3. Results and Discussion

### 3.1. Soil’s Physical and Chemical Properties

**Figure 1:**
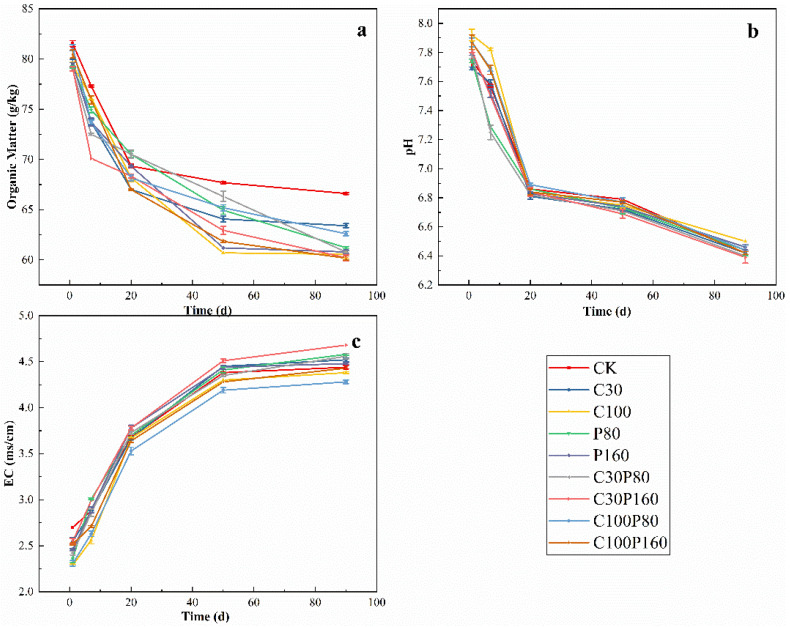
Physicochemical properties of soil, (**a**) organic matter, (**b**) pH, and (**c**) EC.

In the whole experimental process, soil organic matter was decomposed and utilized by microbes (*p <* 0.05). With the increase in culture time, the organic matter contents of soil in different treatment groups showed a downward trend. At the beginning of the experiment, the content of soil organic matter was 80 g/kg, and at the end of the experiment, the content of soil organic matter decreased to 60~65 g/kg (Figure 1a). With the increase of culture days, the pH of each experimental group dropped sharply from 7.7~7.9 to 6.8~6.9 from the 1st to 20th day of the experiment. During the subsequent culture period, the pH decreased slowly to 6.4~6.5 until the end of the experiment (Figure 1b) (*p* < 0.05). Shi et al. suggested that it might be due to the increase of soil base cations release and the decrease of soil exchangeable base cations and cation exchange capacity, resulting in the decrease of soil pH [42]. With the progress of the experiment, the EC of soil increased significantly. From the 1st to the 50th day of the experiment, the EC increased rapidly from 2.3~2.7 ms/cm to 4.0~4.5 ms/cm. In the subsequent culture process, the rising rate of EC slowed down, and the value did not fluctuate significantly (*p* < 0.05). The reason for the increase of EC in the soil might be that the sample contains a lot of inorganic ions [43]. To sum up, the content of soil organic matter and pH decreased continuously during the whole planting process, while the EC value showed the opposite trend. The experimental results verify the conclusion of Antil et al. [44].

### 3.2. ARGs and MGEs

The effects of OTC and Pb application in the soil on ARGs and MGEs were also investigated (Table 4). The abundance of ARGs can reflect the population of drug-resistant bacteria in the soil. The relative abundance of *tetB*, *sul1*, and *sul2* in all samples was found to be high, and the relative abundance order of target genes was as follows: *sul2* > *sul1* > *tetB* > *intl1* > *tetQ* > *intl2* > *sul3*. The results of this study verified the conclusion by Zhu et al. [45], in which the pollutants added to the soil changed the expression of ARGs. More information about ARGs and MGEs can be found in Table 4.

With the change of Pb concentrations added to soil, the abundance of ARGs in soil samples varied remarkably (*p* < 0.05) (Table 4). For the P80 and P160 experimental groups, on the 90th day of the experiment, the relative abundance of *tetB* was noted to be 54.2% and 10.3% of the original abundance, respectively, as compared to the start of the experiment; *tetQ* was 12.6% and 10.7% of the original abundance, respectively; *sul1* was 109.5% and 152.5%; *sul2* was 13.7% and 4.7%; *sul3* was 4.3% and 2.9%; *intl1* was 1.5% and 1.1%; and *intl2* was 77.6% and 98.6% of the original abundance, respectively. At the end of the experiment, the total abundance of MGEs (*intl1* and *intl2*) in the P80 and P160 experimental groups was only 98.6% of that at the beginning of the experiment. On the 90th day of the experiment, the relative abundance of ARGs in the P80 group and P160 group was increased by 0.699-fold and 0.697-fold than that in the CK group, respectively. Similarly, the relative abundance of MGEs in groups P80 and P160 was increased by 0.188-fold and 0.228-fold than that in CK group. In this study, the addition of Pb inhibited the expression of some ARGs, which led to lower enrichment of ARGs. This phenomenon was probably because higher heavy metal content than MIC inhibits the further expression of ARGs [46]. Therefore, excessively high concentrations of Pb in the soil may inhibit the growth and reproduction of microorganisms or even kill them, thereby reducing the expression and transfer of ARGs in the environment.

The relative abundance of ARGs also changed significantly with the addition of OTC in the soil (Table 4). On the 90th day of the experiment, the relative abundance of *tetQ* in C30 and C100 groups was 12.1% and 4.2%; *tetB* in the was 15.2% and 52.2%; *sul1* was 101.8% and 213.8%; *sul2* was 169.4% and 1516.2%; *sul3* was 1.5% and 1.2%; and *intl1* was 0.8% and 2.3% of the original relative abundance, respectively. On the 90th day of the experiment, the total relative abundance of ARGs in the C30 and C100 experimental groups was 82.2% and 84.0% of the CK group, while the total relative abundance of MGEs was 18.8% and 41.0% of the CK group. However, the total relative abundance of *intl2* in the C30 and C100 experimental groups was 20.9% and 25.1% of the CK group, respectively. The addition of OTC in the soil inhibited the spread of *tetQ*, *sul3*, *intl1*, and *intl2* genes and promoted the expression of *sul1* and *sul2* genes. These findings were inconsistent with the previous research studies, which might be due to the fact that the concentration and properties of OTC used in the study are not exactly the same as those used in other experiments [47,48,49]. However, related research studies revealed that adding high concentrations of antibiotics in the soil would adversely affect the structure of the drug-resistant microbial populations; that is, the abundance of soil microorganisms decreases significantly with the increase of OTC concentration [50]. Furthermore, related studies have also demonstrated that an increase in antibiotic concentration gradually decreases antibiotic resistance in corresponding bacterial species [30]. A decrease in the relative abundance of ARGs in the soil cultured with OTC was also observed during the current study, which may also be due to the increased concentration of OTC, which has a toxic effect on the drug-resistant bacteria and hinders the expression of ARGs.

Under the OTC application in soil, Pb treatment further changed the relative abundance of ARGs and MGEs in the soil. On the 1st day of the experiment, the relative abundance of total ARGs in the C30P80 and C30P160 groups were 6.97 times and 4.42 times higher in comparison to group C30, respectively. Further, the total relative abundance of MGEs in the C30P80 and C30P160 groups was 1.31 times and 0.53 times higher than that of group C30, respectively. On the 90th day of the experiment, the relative abundance of total ARGs was 0.99 times and 0.97 times higher than that of the C30 group, respectively. However, the total relative abundance of MGEs on the 90th day of the experiment was 13.80 times and 1.25 times higher than that of the C30 group, respectively. The relative abundance of total ARGs in the C100P80 and C100P160 groups on 1st day of the experiment was 10.56 times and 13.22 times higher than that of the C100 group. The total relative abundance of MGEs was 1.01 and 1.78 times higher than that of the C100 group, respectively. On the 90th day of the experiment, the relative abundance of total ARGs was 1.00 times and 1.55 times higher than that of the C100 group, respectively. The total relative abundance of MGEs was 0.39 and 5.0 times higher than that of the C100 group, respectively. The total relative abundance of ARGs was positively correlated with Pb concentration under OTC-added to the soil. This might be because Pb exposure significantly increased the abundance of MGEs (integrons and inserts) and the overall relative abundance of ARGs [51]. In contrast, this study found that lower concentrations of Pb did not contribute to enhancing the expression of MGEs, resulting in a lower overall relative abundance of ARGs.

In addition, the total relative abundance of ARGs and MGEs decreased with the increase of culture time. This might be due to the combined application of high concentrations of OTC and Pb, which in turn changed the microbial population, inhibited their growth and reproduction, and led to lower expression of ARGs and MGEs. Moreover, Pei, et al. found that *sul* ARGs were higher than *tet* ARGs [52]. These findings demonstrate that the choice of antibiotic has a long-term influence with regard to ARGs.

On the whole, the expression of ARGs was inhibited in different degrees by adding different concentrations of Pb or OTC in the soil. This might be due to the fact that the type of pollutants applied is related to the higher concentration, which has a toxic effect on microorganisms and hinders the spread of ARGs.

### 3.3. The Soil Microbial Community Evolution

**Figure 2:**
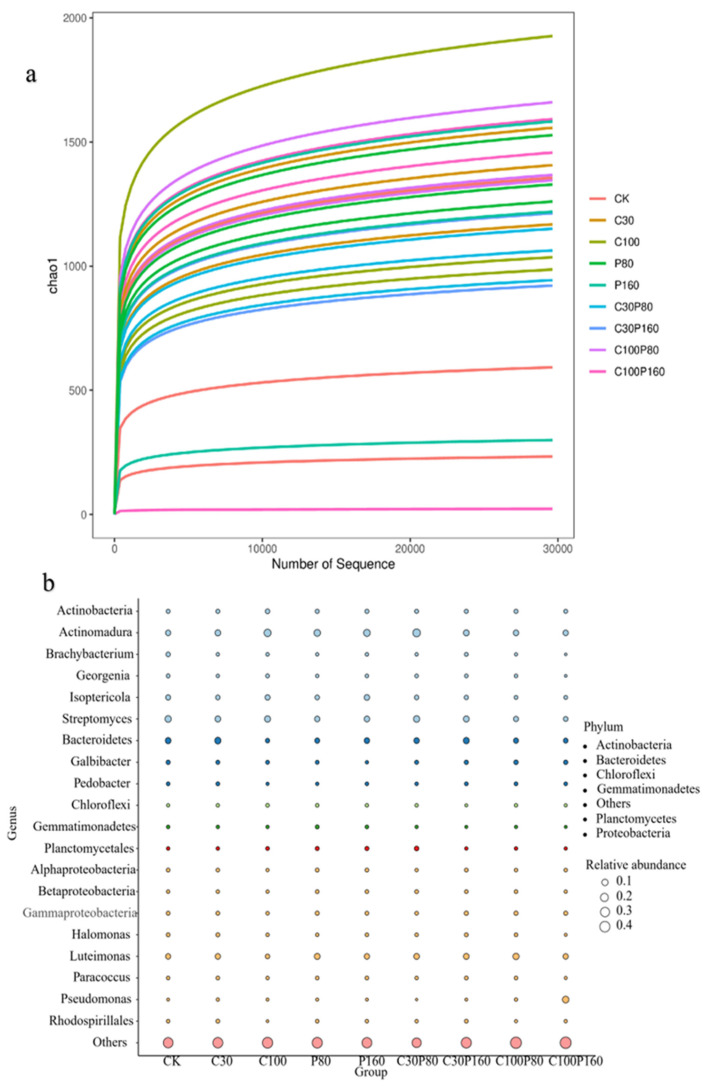
(**a**) Rarefaction curve; (**b**) the bubble plot uses the size and color changes of bubbles to directly reflect the data information of bacterial species annotation and abundant two-dimensional matrix.

Through 16 S rRNA high-throughput sequencing analysis, it was found that the application of Pb and OTC changed the soil microbial community structure. The soil rarefaction curve directly reflected the difference in microbial species diversity and species abundance among different soil samples (Figure 2a). The bubble plot shows genus-level species annotation information and the relative abundance of bacterial species for different sample groups, as well as species annotation information with corresponding phylum (Figure 2b). The bubble plot also shows a relatively high abundance of Actinomadura, Isoptericola, Streptomyces, Bacteroidetes, Galbibacter, and Luteimonas genera in each experimental group, indicating that they were the dominant bacteria under different treatment conditions. It was noted that Pseudomonas only had high relative abundance in the C100P160 group, indicating that *Pseudomonas aeruginosa* had strong adaptability in this environment under high concentrations of Pb and OTC treatments, and was one of the dominant bacteria in this treatment group. After adding Pb and OTC to the soil, the abundance of Isoptericola and Streptomyces was lower than that of the CK group, and this phenomenon became more obvious with the increase of pollutant concentration, which indicated that Pb and OTC had toxic effects on Isoptericola and Streptomyces genus. Compared with Pb, the application of OTC has a more obvious effect on Bacteroidetes. A low concentration of OTC increased the abundance of Bacteroidetes, while a high concentration of OTC inhibited the abundance of Bacteroidetes.

**Figure 3:**
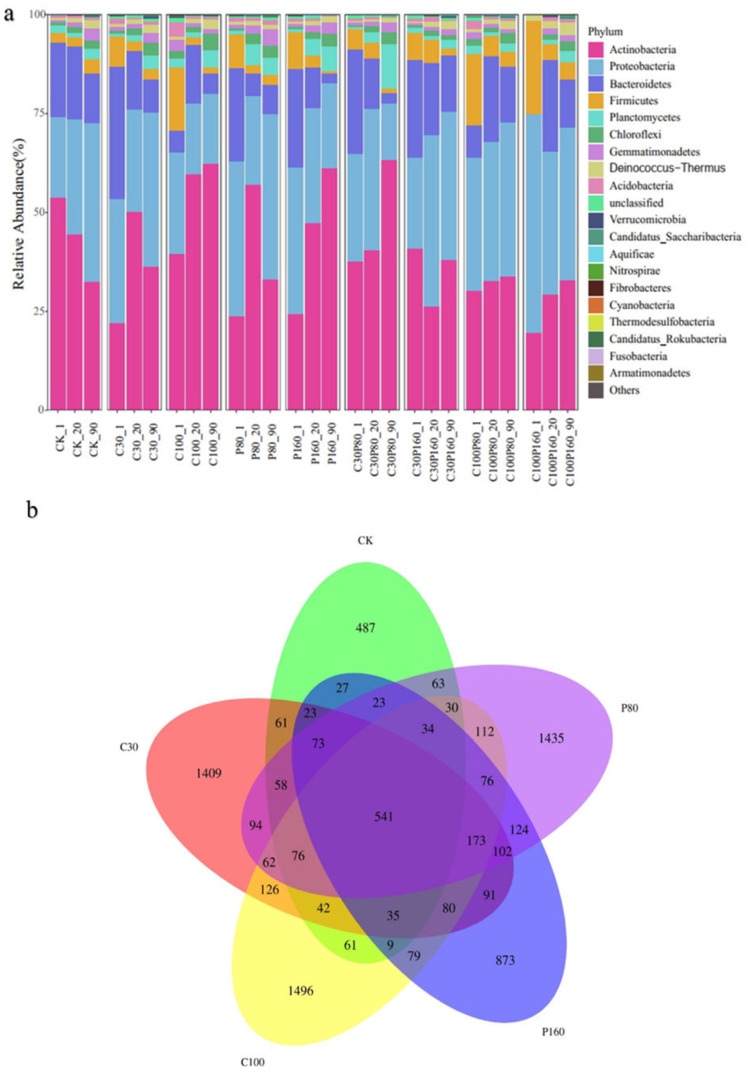
(**a**) The relative population of the top 20 bacteria at the phylum level in the soil; (**b**) Venn diagram showing different OTUs. Note: taking CK-90 as an example, it represents the sample taken by the CK experimental group on the 90th day. The numbers in the Venn diagram represent the number of OTUs.

Figure 3a shows the relative abundance of different bacteria in different samples at the phylum level. Among them, the most dominant bacteria were Actinobacteria (19.59~63.24%), Proteobacteria (14.34~55.33%), Bacteroidetes (0.00~33.37%), Firmicutes (0.65~23.53%), and Planctomycetes (0.00~11.33%). Figure 3a represents 89.03~98.46% of the total bacteria, which is similar to the conclusion of Sardar et al. [53]. Figure 3a also shows that when Pb or OTC was applied alone, high concentrations of Pb or OTC promoted the expression of Actinobacteria, but inhibited the expression of Proteobacteria. On the contrary, when Pb and OTC were applied combined, a high concentration of Pb or OTC inhibited the expression of Actinobacteria, but promoted the expression of Proteobacteria.

The results showed that after OTC or Pb treatment alone, the relative abundance of Actinobacteria increased with the increase of added pollutant concentration. However, when OTC and Pb were applied together, the relative abundance of Actinobacteria decreased with the increase of added pollutant concentration. This indicates that the treatment of a single pollutant may produce resistant strains in microbial communities and enhances the expression of resistant strains. When OTC and Pb are combined, they have toxic effects on bacteria and reduce their metabolism and reproduction, thus affecting their abundance [54]. In addition, it was noted that OTC and Pb treatment had a profound impact on Firmicutes. When OTC or Pb was applied to the soil alone, the abundance of Firmicutes decreased with increasing pollutant concentration, while when OTC and Pb were applied in combination, the abundance of Firmicutes increased with increasing pollutant concentration. The effect of Pb and OTC treatments on the soil microbial community was analyzed by sequencing. Venn diagrams showed 487, 1409, 1496, 1435, and 873 unique operational taxonomic units (OTUs) in the CK, C30, C100, P80, and P160 groups, respectively. Furthermore, 541 OTUs were found to be unique by the five experimental groups (Figure 3b). The results also showed that adding Pb and OTC changed the soil microbial community structure.

**Figure 4:**
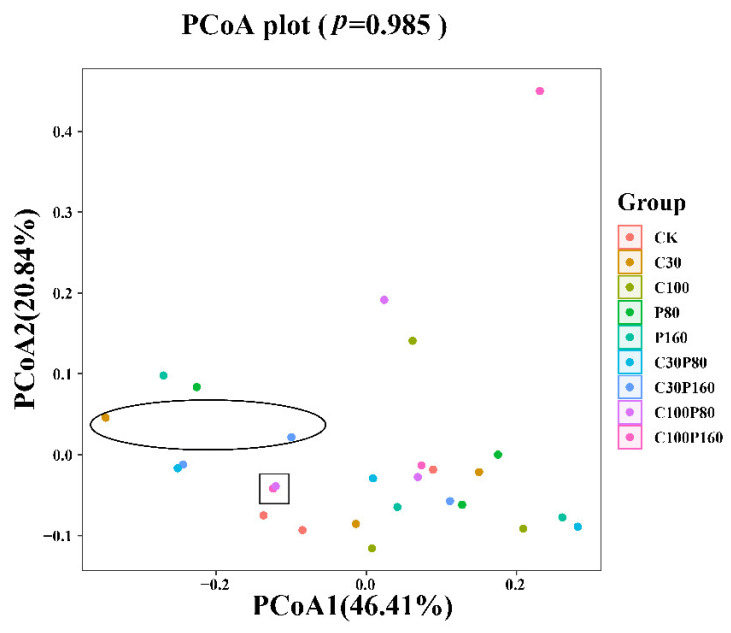
PCoA analysis reflecting the relationship between samples.

Principal coordinates analysis (PCoA) was carried out to estimate the distance matrix and find the best eigenvalues, which can truly reflect the relationship between samples (Figure 4). The different color dots in the PCoA diagram represent different treatment groups, and the distance between the dots indicates the similarity of microbial communities among different samples. PCoA showed a *p*-value of 0.985, as indicated in Figure 4, indicating that the PCoA diagram had high reliability for the interpretation and geometric structures of the samples under different treatment groups. It can be seen from Figure 4 that there were differences in bacterial communities among the samples under different treatment conditions. For example, the purple dot in the square represents the C100P80 treatment group, and the pink dot represents the C100P160 treatment group, which almost coincide with each other, indicating that there was little difference in the microbial community structure between the two treatment groups. On the contrary, the orange dot in the oval represents the C30 treatment group, the blue dot represents the C30P160 treatment group, and the two points are far away, indicating that there is a great difference in the microbial community structure between the two treatment groups.

**Figure 5:**
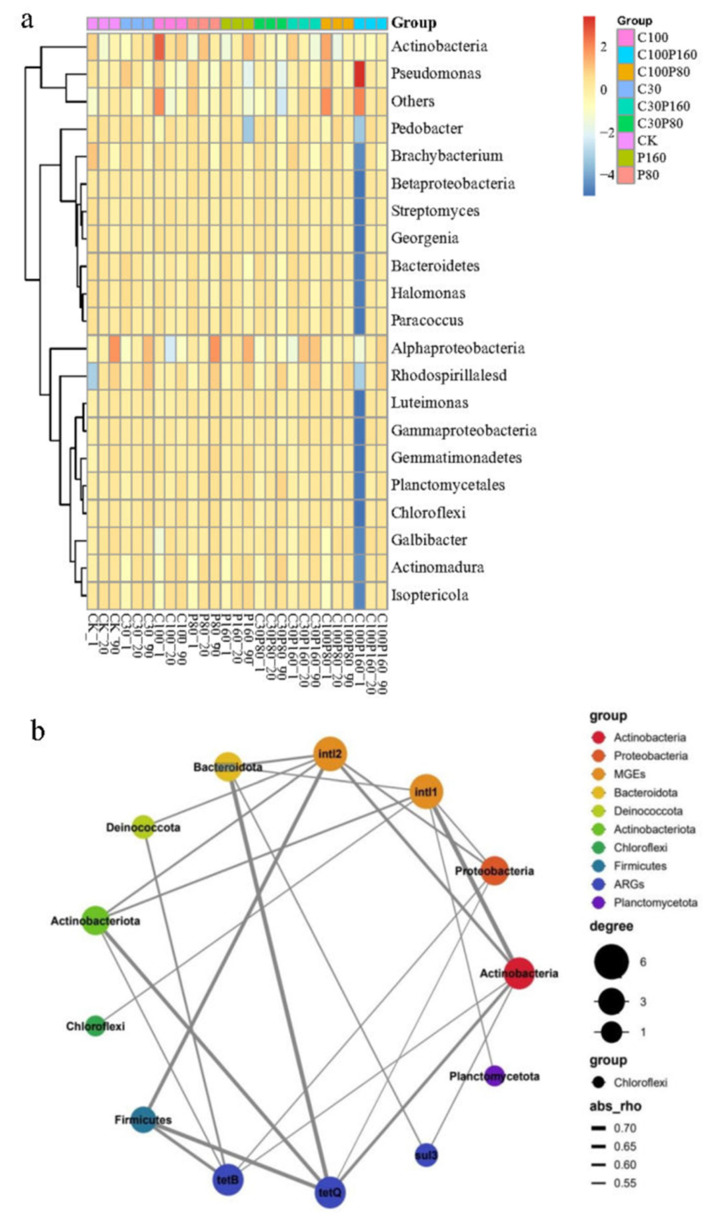
(**a**) Relative abundance heat map of top 20 genera in each treatment; (**b**) network analysis shows the relationship between ARGs, MGEs, and potential host bacteria (including the top 30 bacterial genera).

The relative abundance of Galbibacter, Actinomadura, Isoptericola, and Alphaproteobacteria that were detected was significantly increased at the genus level after alone and combined treatment of Pb and OTC. In contrast, the relative abundance of Actinobacteria, Brachybacterium, and Pseudomonas decreased (Figure 5a). To explain the relevant relationship between the abundance of bacteria and ARGs in the soil samples treated with Pb and OTC, a network analysis was carried out. Network analysis showed a positive relationship among ARGs, MGEs, and potential host bacteria in the soil (Figure 5b). The data shown in Figure 5b includes MGEs, ARGs, and the top 30 bacterial genera. The top 30 bacterial genera were summarized into eight bacterial phylum clades because *sul1* and *sul2* have little correlation with other bacterial species and it was difficult to demonstrate them in the same clade. The network analysis showed that *tetB* was positively correlated with Deinococcota, Firmicutes, Actinobacteriota, Proteobacteria, and Actinobacteria. *TetQ* was positively associated with Bacteroidota, Actinobacteriota, Firmicutes, Proteobacteria, and Actinobacteria. *Sul3* had a positive correlation with Bacteroidota and Actinobacteria. These phylum-level bacteria might be potential repositories of ARGs. Through network analysis, it was found that there were six bacterial genera related to *intl1* and *intl2,* respectively. These bacterial genera were potential MGEs repositories. At the same time, *intl1* and *intl2* might have the same potential host bacteria; they were Bacteroidota, Actinobacteriota, Proteobacteria, and Actinobacteria. Furthermore, among treatment groups such as C30 and C100 treated with OTC alone, a higher abundance of Alphaproteobacteria, Rhodospirillalesd, and Actinobacteria was found due to the increase of ARGs and MGEs. It was suggested that the presence of OTC in the soil would exert pressure on ARGs and resistant bacteria. However, among the other treatment groups, such as P80 and P160, a higher abundance of Actinobacteria, Alphaproteobacteria, Rhodospirillalesd, Gemmatimonadetes, and Isoptericola was also due to the increase of ARGs and MGEs. However, the abundance of Luteimonas did not change much in the P80 and P160 treated groups, which might be due to Luteimonas bacteria not being sensitive to Pb. Proteobacteria accounted for a high proportion among all bacterial genera, with the highest abundance noted in the C100P160 treatment group. From Figure 5b, it was noted that Proteobacteria was positively correlated with *intl1*, *intl2*, *tetB*, and *tetQ* genes. However, high concentrations of Pb and OTC changed the structure of the microbial community, which affected the abundance of ARGs by affecting the expression of MGEs. Network analysis showed that MGEs is not only positively correlated with microbial flora, but also positively correlated with most ARGs (*p* < 0.05). This phenomenon indicated that Pb or OTC treatment might enhance the potential of horizontal transfer of ARGs. According to the network analysis diagram, as shown in Figure 5b, *intl1* and *intl2* genes of bacteria showed a positive correlation with most microbial flora. Furthermore, *Intl1* and *intl2* showed varying degrees of positive correlation with ARGs, which displayed a direct relationship between ARGs and bacteria communities. Luteimonas was a major contributor to the Proteobacteria phylum. The relative abundance of Luteimonas was noted to be higher in C30, C30P160, and C100P160 treatment groups, while a little changed pattern was noted in C100, P80, P160, and C100P80 treatment groups, and decreased abundance was noted in the C30P80 treatment group. The changing trend of Luteimonas under different treatment groups was comparable to that of ARGs. These results reflect the different expression levels of ARGs under different treatment conditions.

### 3.4. Relationship between Physical and Chemical Properties of Soil, Microbial Community, ARGs and MGEs

**Figure 6:**
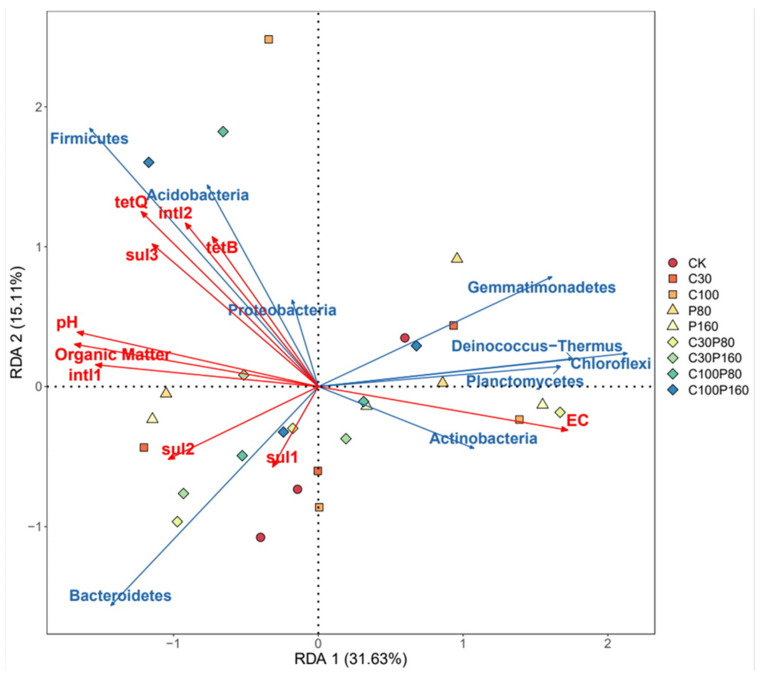
Redundancy analysis (RDA) shows the relationship between treatment groups, major bacterial communities, ARGs, and MGEs.

Soil organic content, EC, and pH affect the ARGs, soil microorganisms’ growth, and reproduction [42]. Redundancy analysis (RDA) was used to analyze the relationships among bacterial community, environmental factors, MGEs, and ARGs. Environmental factors included pH, organic matter, and EC of the soil. The dominant bacterial communities in the samples were Actinobacteria, Proteobacteria, Bacteroidetes, Firmicutes, Planctomycetes, Chloroflex, Gemmatimonadetes, Deinococcus-Thermus, and Aacidobacteria (Figure 6). RDA1 and RDA2 jointly explained 46.74% of ARGs changes. Through RDA analysis, it could be found that ARGs was affected by many factors, and MGEs greatly influenced ARGs. Among the microbial communities, Firmicutes, Acidobacteria, Proteobacteria, Bacterpoidetes, and Bacteroidetes had an inordinate influence on ARGs, suggesting that these microbial communities might be the potential reservoir of ARGs. Microbial communities were greatly influenced by soil organic matter and pH. Soil pH and organic matter content were positively correlated with the relative abundance of ARGs. When soil pH and organic matter content were low, the expression of ARGs was inhibited. The results of this study are consistent with the conclusions of Zhang et al. and Chen et al. [55,56]. Soil acidity also affected the abundance of ARGs, which was mainly because lower pH increased the solubility of pollutants, and a higher concentration of pollutants affected the metabolism of bacteria, which has been confirmed by a previous study [51]. In addition, soil pH and organic matter also contributed significantly to the relative abundance of *intl1* and *intl2* genes, and the contribution of pH and organic matter to the relative abundance of the *intl1* gene was more significant than that of *intl2* gene. Furthermore, EC was negatively correlated with the relative abundance of ARGs and MGEs. MGEs were positively correlated with the expression of *tetB*, *tetQ*, *sul2*, and *sul3* genes. The results of this experiment show that the spread of ARGs might be through the contribution of MGEs [57,58]. It was also confirmed that there was a correlation between antibiotics and the direct selection pressure of ARGs. RDA analysis further showed that *intl1* was positively correlated with all ARGs, while *intl2* had a positive correlation with *tetB*, *tetQ*, *sul2*, and *sul3*, and *intl2* genes were negatively associated with *sul1*. To sum up, *tetB*, *tetQ*, *sul2*, and *sul3* genes could be transmitted not only by *intl1,* but also through *intl2*. ARGs are not only affected by MGEs, but also affected by other environmental pollutants and factors [59,60]. The application of OTC and Pb in this study resulted in a decrease in the number of Bacteroidetes, Proteobacteria, Acidobacteria, and Firmicutes. These bacteria were probably the reservoir bacteria of MGEs, which would further decrease the ARGs. The results of this study show that OTC and Pb inhibited the expression of MGEs, thereby reducing the transfer of most ARGs. MGEs mainly caused the horizontal gene transfer of ARGs, likewise, it was also affected by microbial communities and environmental factors.

## 4. Conclusions

The addition of Pb or OTC resulted in changes in the microbial community structure and a decrease in the relative abundance of Bacteroidetes, Proteobacteria, Acidobacteria, Firmicutes, and total ARGs and MGEs. However, when OTC and Pb were applied in combination, the inhibitory effect of a low concentration of Pb on ARGs was stronger than that of a high concentration of Pb, resulting in a lower abundance of ARGs. Further, network analysis showed that the abundance of ARGs was mainly affected by Actinobacteria, Proteobacteria, and Bacteroidetes. RDA analysis indicated that pH, organic matter, and MGEs significantly contributed to the expression of ARGs. Organic matter content and pH were positively correlated with ARGs abundance, while EC was negatively associated with ARGs and MGEs. Low organic matter content and low pH inhibited the expression of ARGs and MGEs. These conclusions can provide a reference for the transfer of ARGs in the environment, which is the basis of horizontal gene transfer mechanisms.

## Figures and Tables

**Table 1:** Physical and chemical properties of topsoil and chicken manure.

Samples	Moisture Content (%)	pH	Organic Carbon (g/kg)	Electric Conductivity (ms/cm)
Topsoil	10.34	8.38	17.92	1.214
Chicken manure	8.63	7.12	50.24	2.845

**Table 2:** Concentration design of each experimental group.

Groups	CK	C30	C100	P80	P160	C30P80	C30P160	C100P80	C100P160
OTC (mg/kg)	0	30	100	0	0	30	30	100	100
Pb (mg/kg)	0	0	0	80	160	80	160	80	160

**Table 3:** Properties of targeted gene primers.

Gene Type	Genes	Primer	Fragment Length/bp
Tetracycline genes	*tetB* [38]	F: CGAAGTAGGGGTTGAGACGC R: AGACCAAGACCCGCTAATGAA	192
	*tetQ* [39]	F: AGAATCTGCTGTTTGCCAGTG R: CGGAGTGTCAATGATATTGCA	169
Sulfonamides genes	*sul1* [37]	F: CGCACCGGAAACATCGCTGCAC R: TGAAGTTCCGCCGCAAGGCTCG	163
	*sul2* [37]	F: TCCGGTGGAGGCCGGTATCTGG R: CGGGAATGCCATCTGCCTTGAG	191
	*sul3* [40]	F: TCCGTTCAGCGAATTGGTGCAG R: TTCGTTCACGCCTTACACCAGC	143
EMGs	*intl1* [41]	F: CTGGATTTCGATCACGGCACG R: ACATGCGTGTAAATCATCGTCG	473
	*intl2* [37]	F: GTTATTTTATTGCTGGGATTAGGC R: TTTTACGCTGCTGTATGGTGC	166
16 S rRNA	16 S rRNA [37]	F: CCTACGGGAGGCAGCAG R: ATTACCGCGGCTGCTGG	193

**Table 4:** Relative abundance of ARGs of samples from different treatment groups.

Treatments	CK	C30	C100	P80	P160	C30P80	C30P160	C100P80	C100P160
*tetB*	0.0030 ± 3 × 10^−4^ ab	0.0028 ± 1.5 × 10^−4^ b	0.0022 ± 1.5 × 10^−4^ c	0.0014 ± 1.5 × 10^−4^ × 10	0.0021 ± 1.3 × 10^−4^ d	0.0033 ± 3 × 10^−4^ a	0.0022 ± 3 × 10^−4^ c	0.0025 ± 9 × 10^−5^ c	0.0022 ± 1.11 × 10^−4^ c
*tetQ*	2.6150 × 10^−4^ ± 5 × 10^−5^ c	2.2212 × 10^−4^ ± 5 × 10^−5^ c	3.0329 × 10^−4^ ± 5 × 10^−5^ a	2.8212 × 10^−4^ ± 5 × 10^−5^ bc	3.0022 × 10^−4^ ± 5 × 10^−5^ ab	2.4392 × 10^−4^ ± 5 × 10^−5^ c	2.6967 × 10^−4^ ± 5 × 10^−5^ b	2.0902 × 10^−4^ ± 5 × 10^−5^ d	2.1732 × 10^−4^ ± 5 × 10^−5^ d
*sul1*	0.0404 ± 3 × 10^−4^ c	0.0329 ± 6 × 10^−4^ c	0.0422 ± 3 × 10^−4^ b	0.0245 ± 6 × 10^−4^ d	0.0361 ± 3 × 10^−4^ bc	0.0277 ± 6 × 10^−4^ d	0.033 ± 3 × 10^−4^ c	0.0414 ± 3 × 10^−4^ bc	0.0568 ± 0.0015 a
*sul2*	0.0139 ± 2 × 10^−4^ a	0.0114 ± 2 × 10^−4^ ab	0.0036 ± 2 × 10^−4^ c	0.0140 ± 2 × 10^−4^ b	0.0016 ± 2 × 10^−4^ a	0.0156 ± 2 × 10^−4^ c	0.0107 ± 6.6 × 10^−4^ c	0.0041 ± 2 × 10^−4^ bc	0.0157 ± 6 × 10^−4^ a
*sul3*	4.6702 × 10^−6^ ± 0 × 10	6.3394 × 10^−6^ ± 3 × 10^−7^ c	4.8222 × 10^−6^ ± 5.4 × 10^−7^ d × 10	7.4022 × 10^−6^ ± 0 bc	5.7073 × 10^−6^ ± 2.6 × 10^−7^ d	5.6950 × 10^−6^ ± 1.5 × 10^−7^ cd	7.1370 × 10^−6^ ± 2 × 10^−7^ bc	1.2162 × 10^−5^ ± 1.2 × 10^−6^ a	7.3663 × 10^−6^ ± 7 × 10^−7^ b
*intl1*	0.0025 ± 2 × 10^−4^ c	2.6225 × 10-4 ± 2.6 × 10^−5^ f	5.3958 × 10^−4^ ± 5 × 10^−5^ d	2.5314 × 10^−4^ ± 2.5 × 10^−5^ f	2.5633 × 10^−4^ ± 2.5 × 10^−6^ f	0.0071 ± 7.1 × 10^−5^ a	3.3561 × 10^−4^ ± 3.3 × 10^−5^ × 10f	1.2884 × 10^−4^ ± 1.2 × 10^−5^ g	0.0056 ± 2.8 × 10^−4^ b
*intl2*	3.4871 × 10^−4^ ± 0 bc	2.7539 × 10^−4^ ± 2.7 × 10^−5^ c	6.3766 × 10^−4^ ± 3.3 × 10^−5^ a	2.8952 × 10^−4^ ± 2.8 × 10^−5^ c	4.0077 × 10^−4^ ± 2.8 × 10^−5^ b	2.7865 × 10^−4^ ± 2.7 × 10^−5^ c	3.3495 × 10^−4^ ± 3.3 × 10^−5^ c	3.2769 × 10^−4^ ± 3.2 × 10^−5^ c	2.9727 × 10^−4^ ± 2.9 × 10^−5^ c

The different lowercase in the table indicated significant differences between treatments at *p* < 0.05 level.

## Data Availability

The data presented in this study are available on request from the corresponding author.
